# Prospective quantitative evaluation of gait and stance in patients with acute vertigo and dizziness

**DOI:** 10.1007/s00415-025-13191-0

**Published:** 2025-06-12

**Authors:** Hristo Hadzhikolev, Ken Möhwald, Patricia Jaufenthaler, Max Wuehr, Klaus Jahn, Andreas Zwergal

**Affiliations:** 1 German Center for Vertigo and Balance Disorders (DSGZ), LMU University Hospital, LMU Munich, Munich, Germany; 2 Department of Neurology, LMU University Hospital, LMU Munich, Munich, Germany; 3 Department of Neurology, Schoen Clinic Bad Aibling, Bad Aibling, Germany

**Keywords:** Vertigo, Dizziness, Vestibular disorders, Gait imbalance, Vertebrobasilar stroke

## Abstract

**Background:**

Patients with acute vertigo and dizziness often suffer from gait ataxia and postural imbalance. However, detailed and quantitative investigations of gait and stance are largely missing during the acute stage of symptoms.

**Methods:**

This study explores whether assessing objective gait and stance parameters can help differentiate between peripheral and central causes of isolated acute vertigo and dizziness. Patients underwent a standardized protocol within the EMVERT study at the emergency department of LMU University Hospital during the acute stage (on average at 16 h after symptom onset), which included the Timed Up and Go test (TUG), Functional Gait Assessment (FGA), Gait and Truncal Ataxia Index (GTI) and mobile posturography. Patients were categorized into three groups: Acute vestibular strokes (*n* = 56), acute unilateral vestibulopathy (AUVP, *n* = 52) and episodic vestibular disorders (*n* = 92). Outcomes were analyzed using logistic regression models and ROC curves adjusted for age and sex.

**Results:**

We found that patients with AUVP exhibited worse TUG, FGA and GTI scores than those with vestibular strokes or episodic vestibular disorders. ROC curves for TUG, FGA and GTI showed a weak diagnostic accuracy (0.57–0.62) for stroke versus AUVP, which only improved (to 0.75–0.82), if corrected for age and gender. Posturographic sway path was lowest for episodic vestibular disorders, but similar for stroke and AUVP.

**Conclusion:**

Clinical gait and stance tests such as TUG, FGA and GTI do not reliably differentiate central from peripheral etiologies of isolated acute vertigo and dizziness in patients with a mild to moderate burden of symptoms.

**Supplementary Information:**

The online version contains supplementary material available at 10.1007/s00415-025-13191-0.

## Introduction

Acute vertigo and dizziness are the leading symptoms of about 4% of all patient visits to the emergency department (ED). With approximately 13% of the neurological consultations in the ED, it is the third most common complaint after headache and sensorimotor deficits [1–3]. In previous ED cohorts, 4–10% of patients with acute vertigo and dizziness were found to have a stroke as the underlying cause [4]. Diagnostic approaches for patients with acute vestibular syndrome (AVS) are mostly based on the assessment of vestibulo-ocular reflex function and central ocular motor signs established in the head-impulse, nystagmus, test of skew (HINTS) triad [5]. For these parameters, a quantitative assessment by video-oculography has become accessible, enhancing the sensitivity for the differentiation between peripheral and central etiologies [6, 7]. In contrast, assessment of posture and gait in acute vertigo and dizziness in previous studies was limited to course-grained clinical evaluation schemes such as the Gait and Truncal ataxia Index (GTI) [8–10]. These studies claim a high diagnostic accuracy of GTI grades 2 and 3 for vestibular stroke against acute unilateral vestibulopathy [9]. A recent meta-analysis indicated that GTI had only a moderate diagnostic accuracy to detect acute central vestibular disorders with a sensitivity of GTI 2/3 of 70.8%, which was inferior to HINTS [11]. Importantly, quantitative assessments of stance and gait during the acute stage of symptoms are almost completely missing to date.

A more standardized evaluation of postural and locomotor function may be justified by the fact that a relevant proportion of patients with acute vertigo and dizziness do mainly present with gait and stance imbalance and ataxia (recently named acute imbalance syndrome, AIS) [12–14]. Prioritizing the development of a diagnostic algorithm tailored to differentiate the etiologies in patients with a predominant AIS phenotype of AVS in the ED is imperative. This process benefits from a systematic evaluation of stance and gait parameters, which could be both reliable and easily applicable in the acute stage of symptoms (preferably already in the ED).

Given these considerations, we conducted the current study with the aim to prospectively gather objective markers of gait and stance based on established quantitative tests such as Timed Up and Go test (TUG), Functional Gait Assessment (FGA), and mobile posturography in patients presenting with acute isolated vertigo and dizziness (without other central symptoms) during the acute stage. We posed the question, if the extent of impairment reflected by these tests could help separate central from peripheral etiologies of acute isolated vertigo and dizziness.

## Methods

### Protocol approval and patient consent

The study was approved by the Ethics Committee of the University of Munich (ID 57–15) and conducted according to the Guideline for Good Clinical Practice, the Federal Data Protecting Act and the Helsinki Declaration of the World Medical Association (revision of Fortaleza, Brazil, October 2013). All subjects gave their informed, written consent to participate in the study.

### Patient characteristics

Patients with acute isolated vertigo, dizziness, postural imbalance or gait instability, who presented in the ED of the LMU University Hospital in Munich, were prospectively included via the EMVERT (EMergency VERTigo) study [15]. Inclusion criteria comprised of acute onset of symptoms in the last 24 h, which still persisted at arrival to the ED. Patients with clinically proven central disorders (i.e., acute hemiparesis) were excluded from the study and underwent the normal clinical workup. The selection of patients with isolated vertigo and dizziness followed the rationale that this scenario in the ED setting would pose the highest challenge for proper differential diagnosis. Ultimately, 200 patients were included in the current study. We investigated three groups of patients, which were defined by consented International Classification of Vestibular Disorders (ICVD) criteria: vestibular stroke (*n* = 56) [16], acute unilateral vestibulopathy (AUVP, *n* = 52) [17] and episodic vestibular disorders (EV, *n* = 92), including Menière’s disease (*n* = 35) [18], vestibular migraine (*n* = 34) [19] and recurrent benign paroxysmal positional vertigo (BPPV) (*n* = 23) [20].

### Study procedures

All screened patients underwent a workup including a structured medical history with emphasis on previous vertigo/dizziness attacks or accompanying ear or central symptoms. Then a standardized neurological clinical examination was performed. After applying the exclusion criteria, all eligible patients were enrolled within less than 24 h (median duration of 16 h) to the following diagnostic workflow: To assess the grade of gait disorder, patients performed the TUG and FGA. A mobile posturography (Wii Balance Board®, Nintendo, Kyoto, Japan) was used to determine the severity of postural imbalance. The severity of gait and truncal ataxia was characterized using the GTI. The presence of spontaneous nystagmus and additional ocular motor disorders was assessed using video-oculography and video-Head-Impulse-Test (EyeSeeCam®, Fürstenfeldbruck, Germany). The cardiovascular risk profile was evaluated using the ABCD2 score [21, 22]. The Dizziness Handicap Inventory (DHI) was used to quantify the symptom severity [23]. Additionally, all patients underwent a standardized magnetic resonance imaging (MRI) protocol within 7 d after symptom onset (median of 2.1 d) including infratentorial fine slicing to identify or rule out acute central lesions. In cases with a high clinical suspicion of a central etiology but normal initial MRI scan, a second MRI scan was performed at > 3 d from symptom onset to rule out false-negative results due to delayed onset of the diffusion-weighted image (DWI) signal.

### Timed up and go test

The TUG is a diagnostic test for assessing patient´s mobility and fall risk [24, 25]. The patient is asked to stand up unassisted from a seated position, walk to a marked spot 3 m away, turn around, walk back and sit down again. The time required to complete the task is measured by the examining physician. Patients were grouped into three categories based on the severity of impairment adapted from the original publication: No impairment for durations of < 10 s, mild to moderate impairment for durations between 10–29 s and severe impairment for durations ≥ 30 s [24].

### Functional gait assessment

The FGA scale is used to assess walking and balance abilities of patients across various tasks (walking on a flat surface, changing gait speed, walking with horizontal/vertical head turns, quick pivot turns, stepping over obstacles, walking with a narrow base, with eyes closed, backwards, ascending stairs) [26]. It has a maximum score of 30 and a minimum score of 0. A lower score indicates greater impairment of balance. The grade of impairment was evaluated using age-specific thresholds into normal, pathological FGA scores and patients, who scored no points. For patients up to 60 y values ≤ 27, between 60–80 y ≤ 24 and above the age of 80 y ≤ 19 were considered pathological [27, 28].

### Posturography

To measure body sway in the acute phase, we conducted a posturographic analysis using a mobile device (Wii Balance Board®, Nintendo, Kyoto, Japan). We tested four conditions with increasing difficulty levels: bipedal standing/tandem standing with eyes opened/closed. Sway was calculated along the x-axis (medio-lateral) and y-axis (anterior–posterior), and the combined, normalized xy-sway path length (m/min) was analyzed.

### Gait and truncal ataxia index

The severity of gait ataxia was characterized using the GTI, which stratifies the level of impairment into four distinct grades: no gait or truncal ataxia (grade 0), mild to moderate imbalance but can walk independently (grade 1), severe imbalance with standing and cannot walk without support (grade 2) and unable to stand upright unassisted (grade 3) [10].

### Magnetic resonance imaging

An MRI of the brain was performed within a median time of 2.1 d of symptom onset and repeated in some cases with a high clinical suspicion of a central etiology, but DWI being negative on first MRI scan, after > 3 d of symptom onset. The standardized protocol included whole brain and brainstem diffusion-weighted images, fluid attenuated inversion recovery, T2, T2* and 3D-T1, time-of-flight angiography. Two neuro-radiologists assessed all images for the existence of stroke, inflammatory lesions, tumors or other significant pathologies. Stroke lesions most frequently were found in the cerebellum (posterior inferior cerebellar artery (PICA) territory: 32.1%, superior cerebellar artery (SCA) territory: 7.1%), pontomedullary brainstem (30.4%) and mesencephalic brainstem (30.4%). Infarcts in the anterior inferior cerebellar artery (AICA) territory (*n* = 2) were not included in the final statistical analysis because of their hybrid peripheral-central pathophysiology. Their testing results can be found as supplementary material. The infarct size was calculated from lesion maps as described previously [12]. Stroke volumes were relatively small with 4.03 ± 8.03 cm^3^ (mean ± SD).

### Statistical analysis

For the descriptive analysis, means and standard deviations or median values of all parameters (TUG, FGA, combined xy-sway path length) were calculated. Distributions between the patient groups were assessed and evaluated using the chi-square test. We then analyzed TUG, FGA, GTI and sway path for all posturographic test conditions in patients with acute vestibular stroke vs. patients with AUVP vs. patients with EV. One-way analysis of variance (ANOVA) with post-hoc Bonferroni-correction was used for normally distributed data and Kruskal–Wallis test with post-hoc Dunn’s test including Bonferroni-correction for non-normally distributed values. For further analysis, multivariable multinomial logistic regression models with age and gender as covariates were evaluated. For the comparison between AUVP and vestibular stroke, a multivariable logistic regression model with and without these covariates and its corresponding Receiver Operating Characteristic (ROC) curves with evaluation of the Area Under the Curve (AUC) was performed and analyzed (Stata 14.2).

## Results

### Demographic characteristics of the patient cohort

Mean age of the 200 included patients was 58.5 ± 16.2 y with patients in the stroke group being significantly older (66.3 ± 13.1 y) than those with AUVP (53.8 ± 14.5 y) or EV (56.5 ± 17.3 y). Gender distribution in the EV group was nearly balanced, with 53% female patients, whereas male patients were more frequently represented in the stroke (68%) and AUVP (65%) groups. More patients in the stroke group (55%) had ABCD2 scores of ≥ 4 points compared to patients with AUVP (31%) and EV (35%). Chi-square tests revealed significant differences between the three groups in overall ABCD2 score distributions (*p* = 0.005), as well as in the distributions of age, clinical features and duration subscores. The DHI score indicated that patients with stroke were only mildly to moderately affected by vertigo and dizziness compared to AUVP and EV patients (Table 1), which could correspond to the rather small stroke volumes in most patients. Duration from ED admission to advanced gait and stance testing statistically was not different for vestibular stroke (16.5 ± 12.1 h), AUVP (15.1 ± 11.7 h) and EV (14.5 ± 12.3 h). At the time of quantitative gait and stance testing the vast majority of patients was still perceiving vertigo or dizziness with minor non-significant differences between groups (vestibular stroke 87.5%, AUVP 94.2%, EV 84.7%).

**Table 1:** Patient characteristics. Stroke, acute central vestibular lesions; *AUVP* Acute unilateral vestibulopathy; *EV* Episodic vestibular disorder; *DHI* Dizziness Handicap Inventory

Demographic data	Stroke (*n* = 56)	AUVP (*n* = 52)	EV (*n* = 92)	*p*-values*
Age, mean ± SD (years)	66.3 ± 13.1	53.8 ± 14.5	56.5 ± 17.3	*p* = 0.001
Gender, female (n, %)	18 (32.1)	18 (34.6)	49 (53.3)	*p* < 0.05
DHI, mean ± SD (points)	37.0 ± 23.2	60.8 ± 18.0	44.9 ± 20.8	*p* < 0.001
ABCD^2^ Score (*n*, %)				*p* < 0.05
Age				*p* < 0.05
< 60 years (0 points)	18 (32.1)	32 (61.5)	52 (56.5)	
≥ 60 years (1 point)	38 (67.9)	20 (38.5)	40 (43.5)	
Blood pressure				–
≤ 140/90 mmHg (0 points)	18 (32.1)	18 (34.6)	35 (38)	
> 140/90 mmHg (1 point)	38 (67.9)	34 (65.4)	57 (62)	
Clinical features				
Others (0 points)	45 (80.4)	51 (98.1)	91 (98.9)	
Speech impairment (1 point)	7 (12.5)	1 (1.9)	1 (1.1)	
Unilateral weakness (2 points)	4 (7.1)	0	0	
*Duration*				*p* < 0.05
< 10 min (0 points)	0	0	0	
10–59 min (1 point)	9 (16.1)	0	14 (15.22)	
≥ 60 min (2 points)	47 (87.5)	52 (100)	78 (84.7)	
*Diabetes mellitus*				–
No (0 points)	50 (89.3)	51 (98.1)	87 (94.6)	
Yes (1 point)	6 (10.7)	1 (1.9)	5 (5.4)	

*For categorical data chi-square tests were applied, for the other parameters analysis of variance (ANOVA) was used

### Timed up and go test

**Fig. 1:**
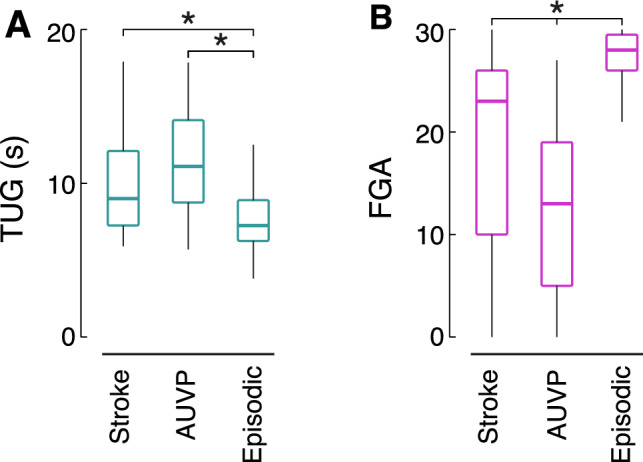
Timed up and Go test (TUG) and Functional Gait Assessment (FGA) outcomes between stroke, acute unilateral vestibulopathy (AUVP) and episodic vestibular disorder patient groups. Higher TUG and lower FGA scores indicate increased levels of impairment. For TUG, there was a significant difference in the overall group comparison (*p* < 0.001): Stroke and AUVP groups differed significantly from the episodic disorders group (*p* < 0.01 and *p* < 0.001, respectively). FGA scores differed significantly among all groups (overall comparison: *p* < 0.001; stroke vs. AUVP: *p* = 0.013; stroke vs. episodic disorders: *p* < 0.001; AUVP vs. episodic disorders: *p* < 0.001). Statistical analysis was performed using the Kruskal–Wallis test and post-hoc Dunn’s test with Bonferroni correction after assessing the data for normality. * *p* < 0.05

In total, 87% of all patients were able to finish the TUG (stroke: 86%, AUVP: 77%, EV: 94%). Only 31% of AUVP patients finished the test in < 10 s. In contrast, 48% of stroke and 75% of EV patients showed no mobility impairment in TUG. In AUVP patients, 44% had mildly to moderately impaired TUG, and 2% showed a severely impaired performance. Conversely, 30% of stroke patients required 10–29 s and 7% > 30 s to finish the task. In the EV group, only 16% of patients showed mild to moderate mobility impairment and 2% a severe impairment (Table 2). Five outliers (*n* = 3 stroke, *n* = 1 AUVP, *n* = 1 EV), who took > 1 min to complete the task, were excluded from the quantitative analysis. Statistical comparison revealed differences between groups (*p* < 0.001). In the post-hoc analysis, stroke and AUVP groups differed significantly from the EV group (*p* < 0.01 and *p* < 0.001, respectively, Fig. 1a).

**Table 2:** Descriptive statistics of clinical scores and tests. *TUG* Timed Up and Go test; *FGA* Functional Gait Assessment; *GTI* Gait and Truncal ataxia Index; *n.a.* not available; Stroke, acute central vestibular lesions; *AUVP* acute unilateral vestibulopathy; *EV* episodic vestibular disorder; the distribution of score results between groups was analyzed using the chi-square test

Descriptive statistics	Stroke (*n* = 56)	AUVP (*n* = 52)	EV (*n* = 92)	*p*-values
TUG (n, %)				*p* < 0.001
< 10 s	27 (48.2)	16 (30.8)	69 (75.0)	
10–29 s	17 (30.4)	23 (44.2)	15 (16.3)	
≥ 30 s	4 (7.1)	1 (1.9)	2 (2.2)	
n.a	8 (14.3)	12 (23.1)	6 (6.5)	
FGA (n, %)				*p* < 0.001
normal	15 (26.8)	2 (3.9)	46 (50.0)	
pathological	22 (39.3)	28 (53.8)	18 (19.6)	
0 points	4 (7.1)	7 (13.5)	0	
n.a	15 (26.8)	15 (28.9)	28 (30.4)	
GTI (n, %)				*p* < 0.001
0 no	26 (46.4)	15 (28.9)	69 (75.0)	
1 mild to moderate	17 (30.3)	23 (44.2)	15 (16.3)	
2 severe	3 (5.4)	4 (7.7)	2 (2.2)	
3 unable to stand unassisted	7 (12.5)	5 (9.6)	0	
n.a	3 (5.4)	5 (9.6)	6 (6.5)

### Functional gait assessment

The FGA was successfully completed in 71% of all patients (stroke: 73%, AUVP: 71%, EV: 70%). In the AUVP group, only 4% of patients achieved normal age-matched test results, while 14% were severely affected (0 points). By contrast, the rate of age-adjusted normal scores was significantly higher in the stroke and EV groups (stroke: 27%; EV: 50%). Only 7% of stroke patients and none in the EV group scored 0 points (Table 2). Statistical comparison revealed group differences (*p* < 0.001) with all pairwise comparisons reaching significance (stroke vs. AUVP: *p* = 0.013; stroke vs. EV: *p* < 0.001; AUVP vs. EV: *p* < 0.001, Fig. 1b).

### Gait and truncal ataxia index

**Fig. 2:**
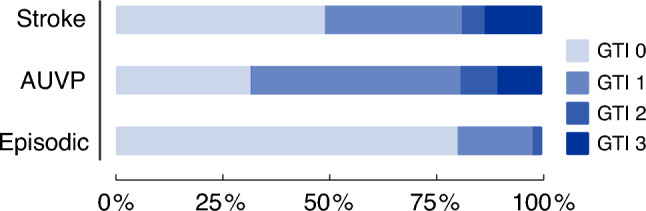
Gait and Truncal ataxia Index (GTI) comparison between groups. There were significant differences on an overall group level (*p* < 0.001) with higher GTI scores in the stroke and AUVP groups (stroke vs. episodic: *p* < 0.001; AUVP vs. episodic: *p* < 0.001). Statistical analysis was performed using the Kruskal–Wallis test and post-hoc Dunn’s test with Bonferroni correction after assessing the data for normality. Stroke, acute central vestibular lesions; AUVP, acute unilateral vestibulopathy; Episodic, episodic vestibular disorder

The severity of gait and truncal ataxia was assessed in 93% of patients. No signs of ataxia (grade 0) were observed in 29% of patients in the AUVP, 46% of stroke patients and 75% of patients in the EV group. 8% of AUVP and 5% of stroke patients displayed a severe grade of ataxia (2), in contrast to 2% of patients in the EV group. A GTI grade of 3 was found in stroke in 13% compared to 10% in AUVP and 0% in EV (Table 2). The comparison of GTI among groups revealed differences (*p* < 0.001) with lower GTI scores in the EV group (stroke vs. EV: *p* < 0.001; AUVP vs. EV: *p* < 0.001) (Fig. 2).

### Posturography

Posturographic assessment in the normal stance with eyes opened condition could be completed by 85% of patients. As test conditions increased in difficulty, completion rates declined progressively, reaching 69% in the tandem stance/eyes closed condition. Statistical comparisons revealed higher sway path values in stroke and AUVP compared to EV patients during normal stance with eyes opened (*p* < 0.001) or closed (*p* < 0.001) as well as during tandem stance with eyes opened (*p* < 0.01), but not during tandem stance with eyes closed (Table 3).

**Table 3:** Sway path (m/min) during different test conditions. Patients with episodic vestibular disorders showed significant differences compared to the stroke and AUVP groups in the first three conditions

	Sway path (m/min) Median (IQR, n)			
Test conditions	Stroke	AUVP	EV	*p*-values*
Normal, eyes opened	1.01 (0.71, 42)	1.02 (0.83, 42)	0.69 (0.42, 85)	*p* < 0.001
Normal, eyes closed	1.25 (1.51, 41)	1.36 (1.83, 42)	0.96 (0.72, 85)	*p* < 0.001
Tandem, eyes opened	3.14 (1.41, 38)	3.00 (2.72, 35)	2.40 (1.73, 78)	*p* < 0.01
Tandem, eyes closed	4.42 (1.83, 36)	4.55 (3.89, 31)	4.55 (2.39, 71)	–

*Statistical analysis was performed using the Kruskal–Wallis test and post-hoc Dunn’s test with Bonferroni correction after assessing the data for normality. Normal, normal stance; Tandem, tandem stance; Stroke, acute central lesions; *AUVP* Acute unilateral vestibulopathy; *EV* Episodic vestibular disorder; *IQR* absolute interquartile range

### Regression and ROC curve evaluation

**Fig. 3:**
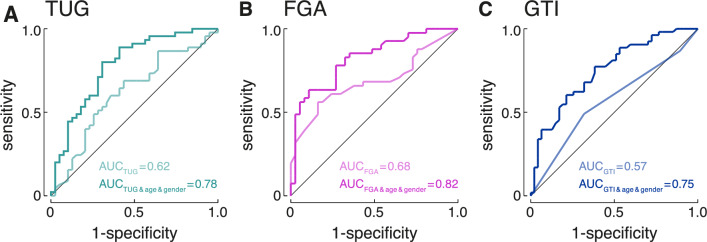
Receiver operating characteristic (ROC) analysis with evaluation of area under the curve (AUC) with and without considerations of age and gender to differentiate stroke and acute unilateral vestibulopathy (AUVP) groups

Multivariable multinomial logistic regression models, adjusted for age and gender, were used to further analyze TUG and FGA outcomes across the three groups. With respect to the TUG, the findings remained consistent with the initial analysis, revealing differences between the EV group and both the stroke and AUVP groups (stroke vs. EV, *p* = 0.01, coefficient −0.15, 95% CI [−0.26, −0.04]; AUVP vs. EV, *p* < 0.001, coefficient −0.24, 95% CI [−0.35,−0.12]). The comparison between stroke and AUVP showed a trend but did not reach statistical significance (*p* = 0.055). In line with the previous results, the FGA showed differences between all groups (stroke vs. AUVP, *p* = 0.002, coefficient −0.08, 95% CI [−0.13, −0.03]; stroke vs. EV, *p* = 0.001, coefficient 0.12, 95% CI [0.05,0.18], AUVP vs. EV, *p* < 0.001, 0.20, 95% CI [0.13,0.27]). In the logistic regression models comparing only stroke to AUVP patients, ROC AUC analyses of TUG, FGA and GTI showed only limited discriminative power (TUG 0.62; FGA 0.68; GTI 0.57). However, after adjusting for age and gender, AUC values increased notably (TUG 0.78, FGA 0.82, GTI 0.75) (Fig. 3).

## Discussion

To our knowledge, this prospective study is the first to comprehensively assess quantitative stance and gait parameters in a large cohort of patients with acute isolated vertigo and dizziness in the acute stage of 24 h after symptom onset. None of the stance and gait tests had a high diagnostic accuracy for the differentiation between peripheral and central etiologies of acute isolated vertigo and dizziness. Contrary to previous studies, the GTI scale did not differ between AUVP and vestibular stroke patients in our cohort. The most likely explanation for this discrepancy may be the different study settings, which led to inclusion of more mildly affected patients in the current study. In general, patients with AUVP displayed more severe gait and stance impairments than patients with vestibular stroke and EV. Taken together, the diagnostic utility of standardized assessment of gait and stance using clinical scales such as TUG, FGA, GTI or quantitative tests such as mobile posturography appears limited in the acute care setting for patients with isolated vertigo and dizziness. Major reasons for this may be that these tests can only be applied in patients with a moderate symptom burden and are quite time-demanding, which limits their routine application in the hyperacute symptomatic stage in the ED. Further limitations may be attributed to the non-negligible influence of age on gait and stance function, as well as inter-individual variability in baseline gait performance. From a practical standpoint the evaluation of stance and gait in the ED setting should be performed with the aim to detect patients with a predominant phenotype of acute gait or truncal ataxia (AIS), which can be efficiently delivered by testing truncal control in sitting position and during standing with eyes open and closed on different stand width (i.e., Romberg and tandem Romberg’s test), as well as testing walking on short distances.

### Diagnostic value of stance and gait assessment for the differentiation of vestibular stroke vs. AUVP

The diagnostic value of assessing gait and truncal ataxia for distinguishing between central and peripheral etiologies of acute vertigo and dizziness has been evaluated in a few previous studies [8, 9, 29]. Carmona et al. investigated 114 patients with AVS, where GTI 2/3 scores had a 93% sensitivity and 61% specificity to detect vestibular stroke in AICA/PICA territory [8]. A recent systematic review and meta-analysis indicated that GTI might be helpful in the clinical workup of patients with AVS, but is inferior compared to HINTS [11]. The advantages of GTI evaluation may lie in its ease of application in the ED, and its relevance for patients with AIS.

In our prospective study, patients with an AUVP performed worse on clinical assessment of gait and stance indicating more severe functional impairment compared to patients with acute vestibular stroke. Consistently, the AUVP group had the highest DHI scores, indicating the greatest perceived handicap. In contrast, patients with vestibular stroke had the lowest DHI scores. These findings challenge the common assumption that a more severe impairment of stance and gait is more indicative of stroke than of peripheral cause in acutely dizzy patients [8, 9]. There are several factors that might contribute to these divergent results: (1) The inclusion criteria of the current study may have led to the selection of patients with a more moderate symptom burden. This especially applies for the patients with vestibular stroke, who mainly had rather small stroke volumes. It can be suggested that patients with an inability to sit or stand unassisted refrained from taking part in this study or were considered ineligible to take part in the more demanding gait and stance tests. This does result in a certain selection bias. On the other hand, patients who are unable to sit or stand unassistedly due to a central lesion often will show additional central clinical signs (such as hemiataxia or dysarthria). Thus, those patients would have been excluded in the current study. (2) Due to logistic demands, testing of gait and stance was not done in the hyperacute stage of symptoms, but within 16 h at median. While the vast majority of patients in all subgroups still were symptomatic at this time, they may have passed the peak of symptoms already. (3) Furthermore, there were additional discrepancies of patient characteristics in the current compared to previous studies. In recent studies, patients with AICA strokes were commonly categorized within the central etiology group [8]. However, occlusion of the AICA leads to ischemia of the cerebellum and labyrinth, resulting in a mixed central-peripheral clinical presentation [30]. For this reason, AICA strokes were not included in our study. Previous studies often compared patient subgroups, which varied in cofactors that potentially affected the clinical assessment of stance and gait [8, 31]. For example, stroke patients are on average older than patients with peripheral etiologies of acute vertigo and dizziness [32–35]. This was also observed in the current study with a mean age difference of 12.5 y between the stroke and AUVP groups. As the prevalence of gait and stance dysfunction increases markedly with age [36, 37], failure to adjust GTI analyses for age may lead to considerable bias, particularly inflating scores in the stroke group. Further confounders for gait assessment may be the varying degrees of comorbidities in the stroke and AUVP groups. Vestibular stroke patients do have a higher prevalence of cardiovascular risk factors such as diabetes, atrial hypertension or obesity, which may contribute to reduced gait capacity and increased impairment due to comorbidities such as polyneuropathy, arthrosis or vascular encephalopathy. In contrast, such gait-affecting comorbidities are uncommon in patients with AUVP or EV. Admittedly, these factors will not be sufficient to explain the differences in AUVP and stroke subgroups in the current study.

However, all these considerations highlight an important limitation of gait and balance assessment in acute vertigo and dizziness: In the hyperacute symptomatic phase, gait and stance assessment in the ED is typically performed without reliable information on the patient’s preexisting gait impairments. For instance, a patient with a severe and chronic gait disorder, who develops AUVP, is more likely to be categorized as GTI grade 3 and to score worse on TUG and FGA.

Consequently, the diagnostic accuracy of clinical tests is limited without adjustment for factors such as age, gender, comorbidities, and baseline gait impairment – yet such adjustments are not practical in the ED. This also applies for instrumented assessments such as the posturography, which are time-consuming and hard to establish in the hyperacute stage with high dropout rates – particularly for more challenging test conditions and in more severely affected patients. Moreover, posturography reveals only minor differences between stroke and AUVP patients on a group level, and even less so on an individual patient level.

### Diagnostic value of gait and stance testing in the acute stage of EV

In our study, EV patients generally had a better performance on gait and stance tasks compared to stroke and AUVP patients. More concretely, EV patients never displayed GTI grade 3 and only rarely GTI grade 2 (Fig. 2). Accordingly, they took significantly shorter times to complete TUG and had higher FGA scores. It may be that part of this findings can be attributed to a decay of symptoms at the time of testing, which is due to the relatively short duration of attacks in these disorders (e.g., a few hours in Menière’s disease or vestibular migraine). For clinical practice, normal age-corrected values for TUG and FGA accompanied by GTI scores of 0/1 more likely suggest a benign EV.

### Limitations

This monocentric study examined a broad cohort of patients with vertigo and dizziness in the acute stage. However, the inclusion criteria requiring isolated vertigo and dizziness without additional major central neurological signs (e.g., hemiparesis) may have introduced a selection bias toward less severely affected patients with central etiologies. We therefore must consider that patients with vestibular stroke in our cohort were only mildly to moderately affected by their vertigo and dizziness, as reflected in DHI scores. In line, the stroke volumes in our cohort were rather small with a mean of 4 cm^3^, likely due to about 60% of lesions being located in the brainstem. It was shown earlier that lesions ≤ 5 cm^3^ may have a more benign clinical course [38, 39]. We acknowledge that patients with a large-vessel occlusion for example of the distal vertebral artery, proximal PICA or basilar artery may be more severely affected by truncal ataxia or gait imbalance. Only 6 stroke lesions in the current study accounted for this subgroup (with lesions ≥ 10 cm^3^). This may mainly explain the difference between our and previous results. Nevertheless, large-vessel occlusions rarely present with isolated vertigo or dizziness, as they are more commonly accompanied by additional central focal neurological signs, such as dysarthria or hemiataxia, which aid in diagnosis.

Another limitation is the relatively high rate of missing data, especially in the 10-items FGA scores, which may be attributed to the time constraints and patients’ exhaustion. Assessment of FGA may therefore be less practical in the ED than TUG/GTI evaluation. Consequently, the risk of a systematic dropout of patients with a more severe impairment of posture and gait must be considered. For the current study, however, the percentage of missing data across different gait and balance tests was similar in the stroke and AUVP groups. We therefore do not consider missing data as a major confounder for our study outcomes.

Lastly, the quantitative testing of stance and gait in the current study was done with a median duration of 16 h from ED admission. While the majority of patients still reported symptoms at that time, it could be that the peak of their clinical signs already had passed and some were already in the state of postural recovery. Testing in the hyperacute stage would have been desirable, but is hardly feasible in the busy environment of an ED.

## Conclusions

This prospective study of patients with acute and isolated vertigo and dizziness reveals substantial limitations of standardized clinical gait and stance assessment in distinguishing central from peripheral disorders at the individual patient level. Age, comorbidities and premorbid posture and gait impairments are likely to confound the diagnostic accuracy of clinical gait and stance tests in the hyperacute stage. In addition, standardized assessment of gait and stance function are time-consuming and may be affected by patient exhaustion, motion intolerance or psychological factors such as fear of falling. Contrary to previous studies, we found a more severe gait and stance impairment in patients with AUVP compared to vestibular stroke. However, it must be acknowledged that the vestibular stroke lesions in the current study were rather small with only a moderate burden of symptoms. More extensive lesions due to large-vessel occlusion will expectedly show a higher degree of posture and gait impairment in vestibular stroke.

From a practical perspective testing of stance and gait in the hyperacute stage of vertigo and dizziness in the ED routine setting mostly follows the purpose to detect patients with a predominant phenotype of acute imbalance syndrome. It can be best done by clinical assessment of truncal ataxia in a sitting position, postural control with eyes open and closed on normal and tandem stance (Romberg/tandem Romberg test) and a judgement, if a patient could walk unassistedly a few meters distance.

## Supplementary Information

Below is the link to the electronic supplementary material.

Supplementary file1 (DOCX 15 KB)

## Data Availability

Anonymized data will be shared upon reasonable request of qualified researchers to the corresponding author.
