# Supplementary material for: Prospective quantitative evaluation of gait and stance in patients with acute vertigo and dizziness
Source: J Neurol. 2025 Jun 12;272(7):458. doi: 10.1007/s00415-025-13191-0 (PMC12162741; doi:10.1007/s00415-025-13191-0)
Supplement: Supplementary file 1 — Supplementary file1 (DOCX 15 KB) [file 415_2025_13191_MOESM1_ESM.docx]

Characteristics of the patients with AICA syndrome:

| **Test conditions** | **Patient 1: 74y old man** | **Patient 2: 82y old man** |
| --- | --- | --- |
| Posturography: Normal, eyes opened | 1.05 | 1.6 |
| Posturography: Normal, eyes closed | 1.55 | n.a. |
| Posturography: Tandem, eyes opened | 3.50 | n.a. |
| Posturography: Tandem, eyes closed | n.a. | n.a. |
| GTI grade | 2 | 3 |
| TUG (s) | 28 | n.a. |
| FGA (points) | 4 | 0 |
